# Rupture of Unscarred Uterus With Intestinal Prolapse From Vagina Following Criminal Abortion

**DOI:** 10.7759/cureus.10601

**Published:** 2020-09-22

**Authors:** Rimpi Singla, Nayana Gaba, Anil L Naik, Anju Singh

**Affiliations:** 1 Obstetrics and Gynaecology, Postgraduate Institute of Medical Education and Research, Chandigarh, IND; 2 General Surgery, Postgraduate Institute of Medical Education and Research, Chandigarh, IND

**Keywords:** illegal abortion, criminal abortion, intestinal prolapse, uterine perforation, instrumentation

## Abstract

A 21-year-old unmarried and primigravida female indulged in criminal abortion at 18 weeks of gestation with the help of a village midwife. Instrumentation was done, and it led to uterine perforation with prolapse of 200 cm of small bowel through vagina. She was managed with resection of 160 cm of necrotic small bowel, repair of the uterine defect, and end jejunostomy, which was anastomosed with distal ileum three months later. This case highlights the risks of illegal abortion and the primitive societal mindset that forces unmarried women to resort to such means.

## Introduction

Criminal abortion is not infrequent in India, and medical help is not always sought [1]. The matter is complicated by archaic societal mindset that stigmatizes women who deviate from the usually established norms regarding marriage and pregnancy as conceiving before marriage is considered a taboo. These abortions are usually fostered by midwives who do not possess any formal training. Instrumentation and use of uterotonic drugs by unskilled personnel place the patient at risk of mechanical complications, and poor hygiene leads to infections. This case highlights the hazards of such practice and represents one of the extraordinary scenarios which an obstetrician can face. There is a need for dissemination of knowledge of legality and risks of criminal abortions along with socioeconomic empowerment of women.

## Case presentation

**Figure 1:**
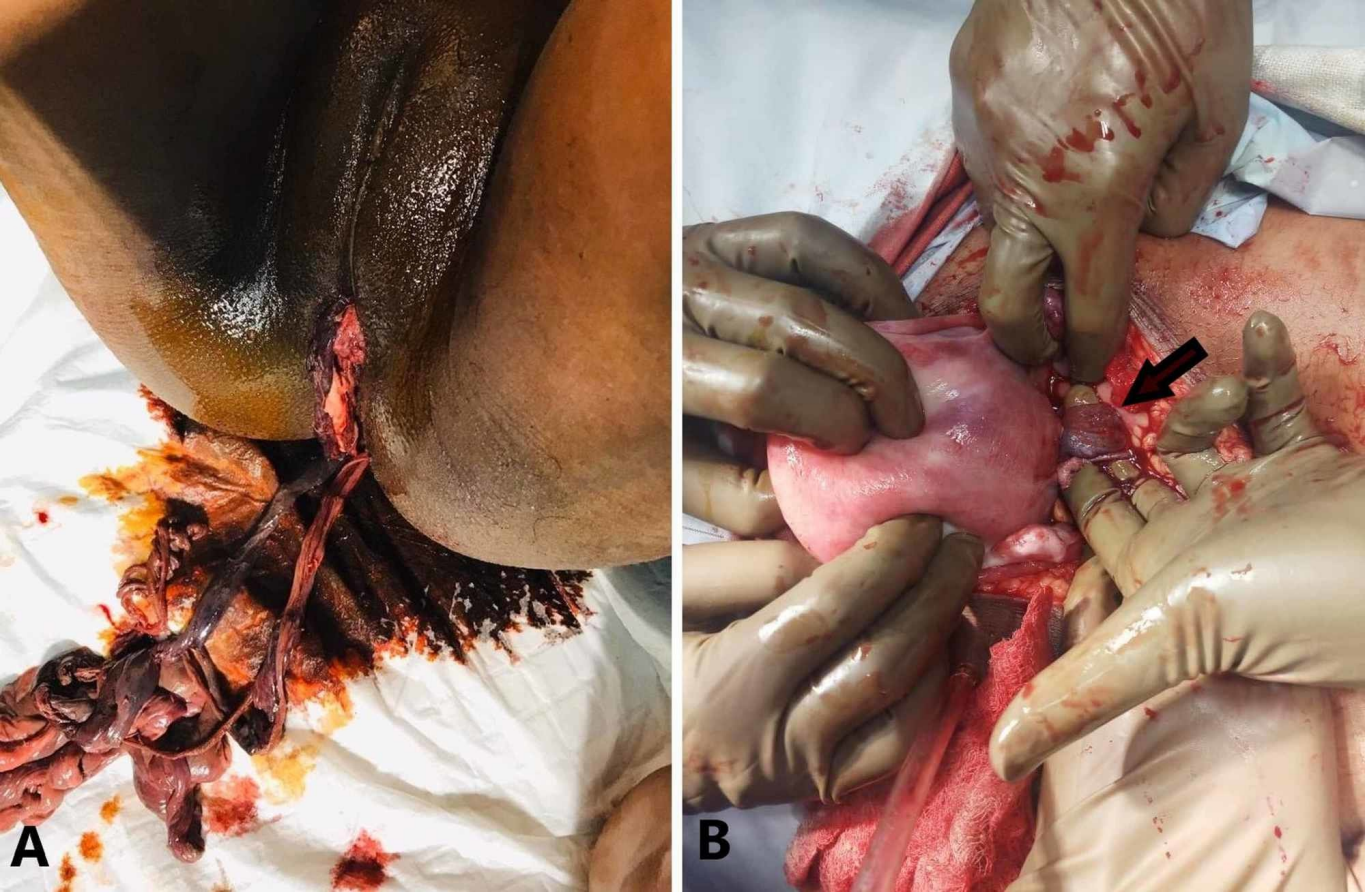
(A) Part of small bowel that had prolapsed from vagina. (B) The site of entry into the uterus (arrow) seen after laparotomy.

**Figure 2:**
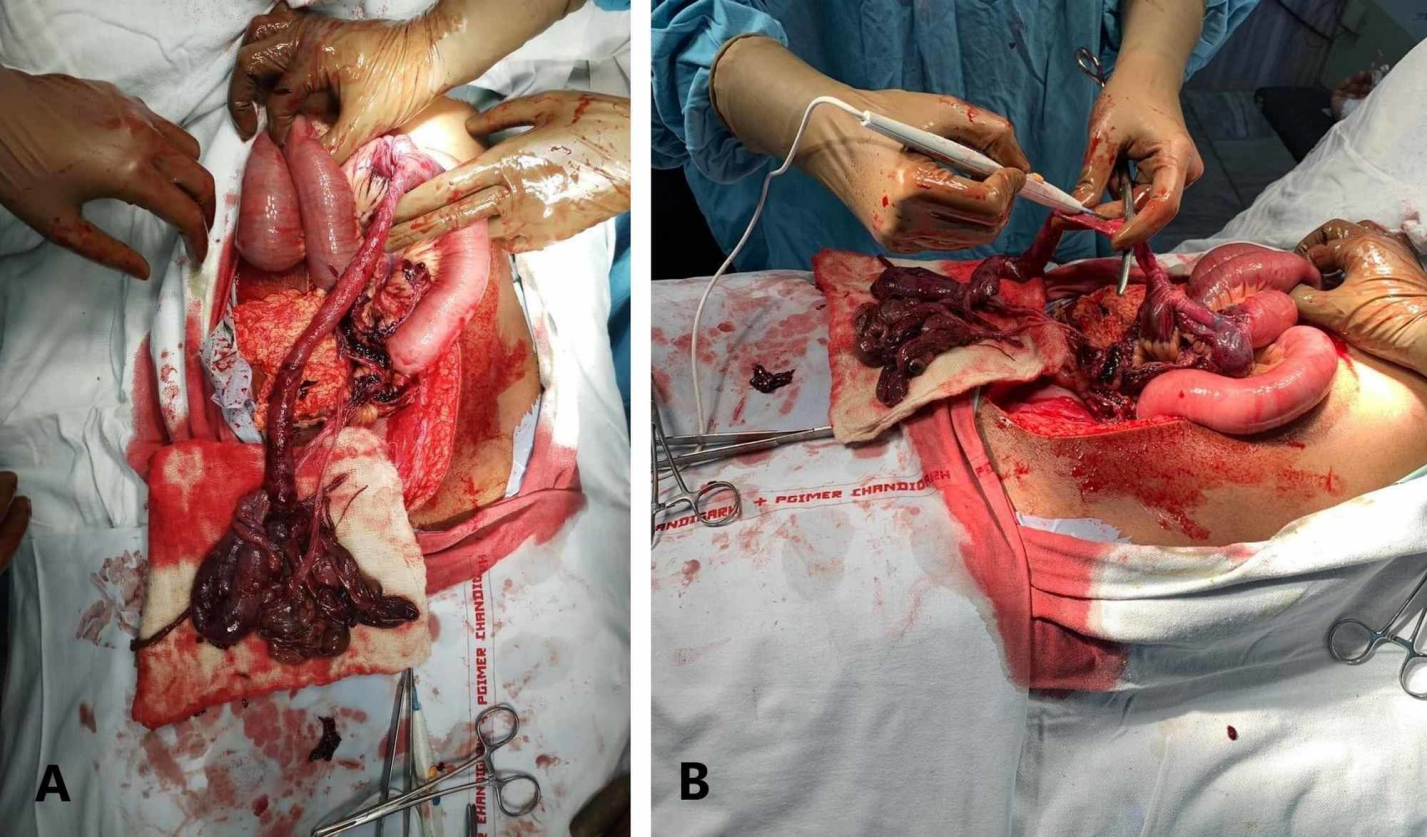
(A) The prolapsed bowel that was pulled back through the uterine defect was gangrenous and detached from mesentery. (B) Resection was done at the junction of gangrenous and viable bowel.

**Figure 3:**
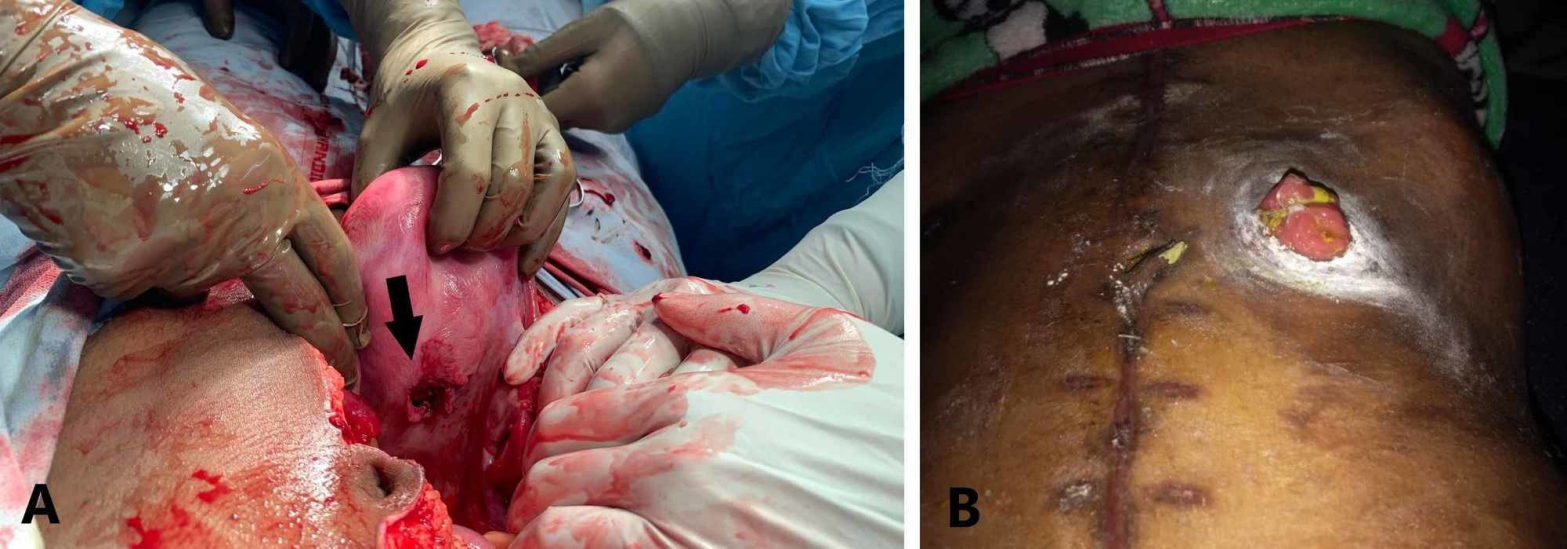
(A) The defect seen on the posterior wall (arrow) of uterus. (B) Jejunal stoma seen post-operatively.

A 21-year-old unmarried female, residing in a village in North India, secretly indulged in illegal abortion at 18 weeks of gestation by consuming abortion pills that she had procured from a local pharmacy. Since the fetus was not expelled, she visited a village midwife the next day, who delivered the baby by instrumentation. Few hours later, she had bouts of cough that led to prolapse of intestine from the vaginum. She was taken to a local hospital from where she was referred to our center. The length of prolapsed bowel increased every time she coughed, strained, or got up from lying position. She reached our hospital 12 hours after the abortion in a state of shock with two meters of small bowel protruding from vagina (Figure 1). Most of the eviscerated bowel was gangrenous and detached from mesentery. She was pale and drowsy, and had blood pressure of 80/50 mm of Hg with a feeble pulse of 120 per minute. The extremities were cold and clammy. Resuscitation with intravenous fluids was followed by an emergency laparotomy. The prolapsed segment (Figure 2) extended from proximal jejunum to distal ileum (30 cm proximal to the ileo-cecal valve). There was no spillage of intestinal contents in the peritoneum, and a 3 x 3 cm defect was present in the posterior uterine wall (Figure 3). The necrotic small bowel (160 cm) was resected and followed by end jejunostomy. The uterus, which was completely viable, was repaired by suturing. Two units of packed red blood cells were transfused during the surgery. Empirical broad spectrum antibiotics were continued for a week, and oral elemental diet was started on the fourth post-operative day. Rest of the hospital stay was uncomplicated. She underwent a follow-up surgery for stoma closure three months later, and jejunum was anastomosed with the distal ileum.

## Discussion

While unsafe abortion is a preventable cause of maternal mortality, it continues to be a public health challenge, accounting for 13% of pregnancy-related mortality worldwide [2]. Worst complications are faced by women of low socioeconomic status residing in countries with restrictive abortion laws. Uterine rupture is said to occur when there is a breach of all the three layers (endometrium, myometrium, and perimetrium). It has classically been described as a complication occurring in women who have previously undergone a cesarean section, particularly those undertaking vaginal birth after a cesarean section. Spontaneous rupture of an unscarred uterus is rare and was found to occur one in 16,849 deliveries in a study [3]. The other causes of uterine rupture are road traffic accidents, instrumentation, and obstetric maneuvers. 

In review of 15 patients with uterine rupture during pregnancy, the rupture occurred during delivery in 10 cases, prior to labor in three cases, during abortion in second trimester in one case, and in puerperal period in one patient [4]. Of the total study population, eight patients had history of a previous cesarean section, three patients had an unscarred uterus, and four patients had a history of instrumentation. All the patients survived, and three of them required hysterectomy. In another review of 30 cases of small bowel obstruction due to uterine perforation resulting from surgical abortion, 18 patients had vaginal evisceration [5]. The length of bowel resected varied from 30 to 200 cm, and stoma creation was required in only one patient. Although not performed in any of the patients studied, irreparable rupture or uterine gangrene can necessitate hysterectomy. In an unusual case of a covert presentation of uterine and intestinal perforation, the diagnosis was made when a patient passed an intrauterine contraceptive device through her bowel, 12 years after it was placed [6]. She had also developed an unwanted pregnancy in the intervening period, and it was felt that the device had spontaneously passed out of the uterus. The other structure that can prolapse through a uterine perforation is omentum. There are case reports of this occurring after dilatation and curettage [7,8]. Small lesions can be managed by hysteroscopic approach, and larger ones may require open surgery.

Bowel prolapse and perforation are rare but serious complications of surgical abortion. Multiple surgeries may be required that prolongs the hospital stay, increases the financial burden, and leads to long-term sequelae [9]. Terminal ileum and pelvic colon are the most commonly injured parts of bowel due to their anatomic predisposition. Most of the patients require resuscitation, resection, and lavage. Simple closure may be feasible in solitary perforations, but multiple perforations or gangrenous bowel may require segmental intestinal resection followed by primary anastomosis or stoma creation. Such cases are best managed by a multidisciplinary team comprising of surgeons specializing in gynecology and gastroenterology. Injury to the bladder requires involvement of a urologist. It is important to be able to quickly identify the structures that can be salvaged, and devitalized structures need to be removed. Loss of a part of bowel can lead to malabsorption and poor nutritional status. Hysterectomy causes sexual dysfunction and renders the patient incapable of having a child in future [10]. Premature surgical menopause results in vasomotor symptoms (hot flushes) and increased risk of osteoporosis and cardiovascular disease. These factors lead to poor quality of life and increased health risks.

## Conclusions

Criminal abortions carry a very high risk of complications, especially when it involves instrumentation. This case represents one such scenario in which the patient developed uterine rupture with prolapse of a large segment of small bowel. Although she required resection of the prolapsed bowel, timely surgical management helped save her life.
